# Redox modulation of NQO1

**DOI:** 10.1371/journal.pone.0190717

**Published:** 2018-01-03

**Authors:** David Siegel, Donna D. Dehn, Samantha S. Bokatzian, Kevin Quinn, Donald S. Backos, Andrea Di Francesco, Michel Bernier, Nichole Reisdorph, Rafael de Cabo, David Ross

**Affiliations:** 1 Department of Pharmaceutical Sciences, Skaggs School of Pharmacy, University of Colorado Anschutz Medical Campus, Aurora, Colorado, United States of America; 2 Agilent Technologies, Inc., Lexington, Massachusetts, United States of America; 3 Experimental Gerontology Section, Translational Gerontology Branch, National Institutes on Aging, Baltimore, Maryland, United States of America; The University of Texas MD Anderson Cancer Center, UNITED STATES

## Abstract

NQO1 is a FAD containing NAD(P)H-dependent oxidoreductase that catalyzes the reduction of quinones and related substrates. In cells, NQO1 participates in a number of binding interactions with other proteins and mRNA and these interactions may be influenced by the concentrations of reduced pyridine nucleotides. NAD(P)H can protect NQO1 from proteolytic digestion suggesting that binding of reduced pyridine nucleotides results in a change in NQO1 structure. We have used purified NQO1 to demonstrate the addition of NAD(P)H induces a change in the structure of NQO1; this results in the loss of immunoreactivity to antibodies that bind to the C-terminal domain and to helix 7 of the catalytic core domain. Under normal cellular conditions NQO1 is not immunoprecipitated by these antibodies, however, following treatment with β-lapachone which caused rapid oxidation of NAD(P)H NQO1 could be readily pulled-down. Similarly, immunostaining for NQO1 was significantly increased in cells following treatment with β-lapachone demonstrating that under non-denaturing conditions the immunoreactivity of NQO1 is reflective of the NAD(P)^+^/NAD(P)H ratio. In untreated human cells, regions with high intensity immunostaining for NQO1 co-localize with acetyl α-tubulin and the NAD^+^-dependent deacetylase Sirt2 on the centrosome(s), the mitotic spindle and midbody during cell division. These data provide evidence that during the centriole duplication cycle NQO1 may provide NAD^+^ for Sirt2-mediated deacetylation of microtubules. Overall, NQO1 may act as a redox-dependent switch where the protein responds to the NAD(P)^+^/NAD(P)H redox environment by altering its structure promoting the binding or dissociation of NQO1 with target macromolecules.

## Introduction

NAD(P)H:quinone oxidoreductase 1 (NQO1, EC 1.6.99.2) is a homodimeric, flavin-dependent, two-electron reductase with the capacity to reduce a broad range of substrates. In humans NQO1 is expressed at high levels in epithelium, endothelium and adipocytes as well as many solid tumors [1, 2]. NQO1 is unique among cellular reductases because it can utilize a broad range of reducing cofactors including NADH and NAD(P)H [3, 4]. NQO1 has traditionally been labeled as a quinone reductase but new roles for NQO1 are emerging where this enzyme binds to and regulates the stability of many important biological molecules. A number of studies have been published where NQO1 can modulate the levels of critical regulatory proteins including p53, p63, p73, PGC-1α and most recently Hif-1α by protecting these proteins from 20S proteasomal degradation [5–9]. In many of these studies the ability of NQO1 to perform this modulatory role was dependent upon the addition of reduced pyridine nucleotides [6, 7, 10]. In addition, NQO1 was detected in a large screening study designed to identify novel mRNA binding proteins [11]. NQO1 has been shown to bind to the 3’ untranslated region and the coding region of SERPINA1 mRNA, which encodes for the protein α-1 antitrypsin. Similar to what was observed with 20S proteasomal substrates, the binding of NQO1 to SERPINA1 mRNA could be altered by manipulating the pyridine nucleotide redox balance [12].

NQO1 is well suited to modulate its conformation in response to reduced pyridine nucleotide levels since it utilizes a *ping-pong bi-bi* catalytic mechanism where (1) NQO1 binds reduced pyridine nucleotide (2) a hydride transfer occurs from NAD(P)H to enzyme bound FAD (3) the enzyme undergoes a conformational change expelling NAD(P)^+^ from the active site, and (4) a suitable substrate enters the active site and accepts the hydride from FAD to reestablish the oxidized enzyme [13]. This mechanism allows for NQO1 to adopt an oxidized conformation (Enz-FAD) in the absence of reduced pyridine nucleotides or a reduced conformation (Enz-FADH_2_) in the presence of reducing cofactors. The ability of NQO1 to maintain either an oxidized or reduced conformation depends primarily upon the supply of reduced pyridine nucleotides and enzyme substrates.

In this study, we examined whether we could detect a conformational switch in NQO1 in response to alterations in reduced pyridine nucleotide concentrations. We utilized purified recombinant human NQO1 and human cell lines expressing NQO1 to test whether the binding of antibodies to epitopes located on the catalytic core and C-terminal domains could be modulated by the redox state of NQO1.

## Materials and methods

### Reagents

Purified recombinant human NQO1(rhNQO1), FAD, NADPH, NADH, dicumarol, cibacron blue, gelatin (type-A), Triton X-100 and 2-(4-amidinophenyl)-1H-indole-6-carboxamidine (DAPI) were purchased from Sigma-Aldrich (St Louis MO). β-lapachone and n-benzyldihydronicotinamide were obtained from Santa Cruz Biotechnology (Dallas, TX) and the PARP1/2 inhibitor olaparib was purchased from Selleckchem (Houston, TX). Reduced nicotinamide riboside (NRH) was synthesized from NADH as previously described [4]. Purified NQO1was reconstituted in 25mM Tris-HCl containing 250mM sucrose and 5μM FAD aliquoted and stored frozen at -80°C. Recombinant human NQO1*2 was purified from E coli as described previously [14, 15].

### Cell lines

The human bronchial epithelial cell line 16HBE was obtained from Dr. Brian Day, Department of Medicine, National Jewish Health, Denver, CO and grown in DMEM containing 10% (v/v) fetal bovine serum, 100units/ml penicillin and 100μg/ml streptomycin (complete medium). Transformed human bone marrow endothelial cells (TrHBMEC) were obtained from Dr. Babette Weksler, Weill Medical College, Cornell University, New York, NY. Cells were cultured on 0.2% (v/v) gelatin coated plates in DMEM (low glucose) supplemented with 5% (v/v) fetal bovine serum, 3mM L-glutamine, 10mM HEPES, 1% (v/v) BME vitamins (Invitrogen, Carlsbad, CA), 100units/ml penicillin and 100μg/ml streptomycin (complete medium). All cell lines were maintained in a humidified incubator at 37°C with 5% carbon dioxide.

### siRNA-mediated knockdown of NQO1

TrHBMEC (2 x 10^5^) were seeded into individual wells of a 6-well plate containing a single 18mm glass coverslips precoated with 0.2% gelatin in 2ml of complete medium and 24 h after seeding the medium was replaced with 2ml complete medium minus antibiotics for an additional 16h. On-target plus non-targeting siRNA (control) and on-target plus human NQO1siRNA smartpool were purchased from Dharmacon (Lafayette, CO). siRNAs and DharmaFect 4 transfection reagent (Dharmacon) were prepared for transfection (6-well plate) as described by the manufacturer (see: http://dharmacon.gelifesciences.com/uploadedFiles/Resources/basic-dharmafect-protocol.pdf)) For these studies each transfection was carried out using 50nM of siRNA in combination with 2μl of DharmaFect 4 transfection reagent. Cells were treated with siRNA/transfection reagent diluted in DMEM containing 10% FBS (no antibiotics) for 72 h after which the coverslips were removed and processed for immunocytochemistry as describe below.

### Antibodies

Primary antibodies used in these studies were purchased from commercial sources and used as described in S1 Table. Non-specific staining was assessed using purified isotype-matched mouse IgG_1_ (NBP1-97005, Novus Biologicals, Littleton, CO) at a dilution of 1:500 and purified rabbit IgG (I1000, Vector Laboratories, Burlingame, CA) diluted to 1mg/ml in phosphate buffered saline (PBS) and used at a dilution of 1:5000. Alexa fluor 488-conjugated goat anti-mouse IgG (diluted 1:2000), rhodamine red-X-conjugated goat anti-rabbit IgG (diluted 1:1000) and horseradish peroxidase-conjugated goat anti-mouse or -rabbit IgG (diluted 1:7500) secondary antibodies were purchased from Jackson ImmunoResearch Laboratories (West Grove, PA).

### Tryptic digestion of NQO1

Tryptic digestion of purified rhNQO1 was performed in 25mM Tris-HCl, pH 7.4 containing 5μM FAD. Reactions (50μl) contained 2μg rhNQO1 in the absence and presence of 0.2mM NADH. Proteolytic digestion was initiated by the addition of 0.1μg of TPCK-treated trypsin (bovine pancreas, Sigma-Aldrich) for 30 min at 37°C after which 2μl of the above reaction was diluted in 278μl of 3X Laemmli buffer (Boston BioProducts, Ashland, MA), heated to 85°C for 7 min and 20μl was separated by 12% SDS-PAGE (precast Mini-Protean, BioRad, Hercules CA). Proteins were then transferred to a 0.2μm PVDF membrane in 25mM Tris containing 192mM glycine and 20% (v/v) methanol at 150 volts for 2 h at 4°C. Membranes were blocked in 10mM Tris-HCl, pH 7.8, 150mM NaCl and 0.2% (v/v) Tween-20 (TBST) containing 5% (w/v) non-fat dry milk for 1h at 22°C and primary antibodies were diluted in TBST containing 5% non-fat dry milk (see S1 Table, for primary antibody dilutions), and then used to probe membranes for 1h at 22°C. Membranes were washed extensively in TBST followed by the addition of horseradish peroxidase-conjugated secondary antibodies diluted in TBST containing 5% non-fat dry milk for 30 min at 22°C. Membranes were washed extensively with TBST and protein bands were visualized using enhanced chemiluminescence (GE Healthcare Life Sciences, Pittsburgh, PA).

### Immunoprecipitation of rhNQO1

Purified rhNQO1 (0.2μg) was added to 25mM Tris-HCl, pH 7.4 containing 1mg/ml BSA and 5μM FAD (0.5ml final volume) in the presence or absence of 0.2mM reduced pyridine nucleotides or analogs. After 15 min either anti-NQO1 A180 or C-term antibody (2μg) was added for 1 h at 22°C followed by 25μl of protein A/G plus agarose (Santa Cruz Biotechnology) for an additional 30 min. Beads were collected by centrifugation at 5000 rpm for 1 min at 22°C, washed 3 times with 0.5ml of 25mM Tris-HCl, pH 7.4 containing 1mg/ml BSA and 5μM FAD, then once with 0.5ml of 25mM Tris-HCl, pH 7.4. Beads were resuspended in 25μl of 3X Laemmli buffer (Boston BioProducts), heated to 85°C for 7 min and 20μl was analyzed by immunoblot analysis as described above (see Tryptic Digestion of NQO1).

### Non-denaturing PAGE

Purified rhNQO1 was analyzed by non-denaturing polyacrylamide gel electrophoresis in the presence and absence of NADH. For these analysis rhNQO1 (50μg) was diluted in a final volume of 50μl containing 62.5mM Tris-HCl, pH 6.8, 40% (w/v) sucrose, 20μM FAD and 1% (w/v) bromophenol blue in the absence (no additions) or the presence of 1mM NADH (plus NADH) or 60μM dicumarol (plus dicumarol). Samples (10μg/10μl) were loaded into each well of a BioRad 4–20% Mini-Protean precast gradient gel. Electrophoresis was performed using freshly prepared running buffers containing 25mM Tris, 192mM glycine and 5μM FAD (no additions) or with 0.2mM NADH (plus NADH) or 50μM dicumarol (plus dicumarol). Electrophoresis was carried out at 150 volts for 16 h at 4°C. Since NQO1 has an overall positive charge the electrodes were in reverse orientation and the protein was attracted towards the negative electrode. Following electrophoresis protein bands were visualized using Coomassie dye R-250 staining (Imperial Protein Stain, ThermoFisher, Waltham, MA). The purity of rhNQO1 used in these studies was determined by denaturing SDS-PAGE and shown in S1 Fig.

### β-Lapachone treatment and immunoprecipitation of NQO1 from human cell lines

Human cell lines were grown in 100mm tissue culture plates in 10ml complete medium to approximately 75% confluency. Growth medium was replaced with complete medium containing 10μM β-lapachone for the indicated times after which the drug containing medium was removed and the plates washed twice with PBS. The plates were placed on ice and the cells scrapped into 300μl of ice-cold cell lysis buffer (Cell Signaling Technologies). The cell lysate was transferred to 1.5ml microfuge tube then sonicated for 3s on ice. Protein concentrations of the resultant sonicates were determined using the method of Lowry [16] and 0.5mg of cellular protein was transferred to a new 1.5ml tube and the volume was brought up to 0.5ml with cell lysis buffer. Anti-NQO1 C-terminal or A180 antibodies (2μg) was added for 1 h at 22°C followed by 25μl of protein A/G plus agarose (Santa Cruz Biotechnology) for 30 min at 22°C. Beads were collected by centrifugation at 5000 rpm for 1 min at 22°C, washed 3 times with 0.5ml of 25mM Tris-HCl, pH 7.4 containing 1mg/ml BSA and 5μM FAD, then once with 0.5ml of 25mM Tris-Cl, pH 7.4. Beads were resuspended in 25μl of 3x Laemmli buffer, heated to 85°C for 7 min then processed for immunoblot analysis as described above (see Tryptic Digestion of NQO1).

### Measurement of pyridine nucleotide concentrations by mass spectrometry

Intracellular oxidized and reduced pyridine nucleotide concentrations were determined in 16HBE cells treated with β-lapachone. For these studies 16HBE cells were grown to approximately 75% confluency in 100mm plates in 10ml of compete medium and 24 h prior to analysis the medium was replaced with 10ml of fresh complete medium. In studies with olaparib (PARP1/2 inhibitor) cells were pretreated for 30 min with olaparib (1μM) or DMSO (0.01%) in growth medium. Treatments were initiated by removing the growth medium and adding 10ml of complete medium containing β-lapachone (10μM) or β-lapachone (10μM) and olaparib (1μM). After 2 h at 37°C the medium was aspirated, the cells washed once with PBS then placed on ice. Cells were scrapped into 0.5ml of ice-cold methanol (100%) at 4°C then transferred to a precooled 1.5ml microcentrifuge tube and centrifuged at 13,000rpm for 3 min at 4°C. Supernatant (0.2ml) was transferred into two 1.5ml microcentrifuge tubes and the contents were lyophilized under vacuum (speedvac) at 45°C for 1h. For analysis of oxidized pyridine nucleotides one tube was rehydrated with 0.1ml of 50% (v/v) methanol/water and separation was performed using reverse-phase HPLC where 1μl was applied to a Xselect HSS T3 column (2.1 x 100mm, 2.5μm; Waters, Milford, MA) with a column oven temperature of 35°C. Chromatography was carried out using a linear gradient of 0 to 30% methanol over 9 min (buffer A, 0.1% (v/v) formic acid in water; buffer B, 0.1% (v/v) formic acid in methanol) with a flow rate of 0.35ml/min. For analysis of reduced pyridine nucleotides the remaining tube was rehydrated with 25μl of 95:5 0.01M NaOH:acetonitrile and separation was performed using hydrophilic interaction liquid chromatography (HILIC) where 1μl was applied to a Luna NH_2_ column (2 x 100mm, 5μm; Phenomenex, Torrance, CA) with a column oven temperature of 25°C. Chromatography was carried out using a linear gradient of 85% buffer B to 0% buffer B over 3 min then hold at 0% buffer B for an additional 4.6 min (buffer A, 20mM ammonium acetate and 20mM ammonium hydroxide in 95:5 water: acetonitrile, pH 9.6; buffer B, 100% acetonitrile) with a flow rate of 0.3ml/min. Mass spectra were obtained using an Agilent 6410 triple quadrupole mass spectrometer with electrospray ionization (negative mode). Calibration curves for NADH and NAD^+^ were run using authentic standards. Total protein concentrations were determined using the method of Lowry [16] in identically plated and treated cells scrapped into 0.5ml lysis buffer (Cell Signaling Technologies).

### Immunocytochemistry

Immunocytochemical analysis was performed on 16HBE cells seeded onto18 mm glass coverslips or TrHBMEC seeded onto 18 mm glass coverslips precoated with 0.2% (v/v) gelatin in PBS. Unless otherwise stated cells were grown in complete tissue culture medium to approximately 75% confluency then the coverslips were rinsed with PBS and fixed with 4% (v/v) methanol-free formaldehyde (Ted Pella Inc., Redding, CA) diluted in PBS for 12 min at 22°C. Coverslips were rinsed 3 times with PBS then permeabilized with 0.1% (v/v) Triton X-100 diluted in PBS for 12 min at 22°C. Coverslips were rinsed 3 times with PBS then placed in a 6-well dish (1 coverslip/well) and blocked for 1 h (22°C) in 2ml complete tissue culture medium diluted 1:1 with 10mM Tris-HCl, pH 7.4 containing 150mM NaCl and 0.2% (v/v) Tween-20 (blocking buffer). Primary antibodies were diluted in blocking buffer (see S1 Table) and 1ml of antibody containing solution was added to each coverslip for 1 h at 22°C. After exposure to primary antibodies coverslips were washed extensively with 10mM Tris-HCl, pH 7.4 containing 150mM NaCl and 0.2% (v/v) Tween-20 (TBST). Secondary antibodies were diluted in blocking buffer (1ml) containing DAPI (1μg/ml) and were added for 30 min at 22°C. Coverslips were again washed extensively in TBST, dipped in ddH_2_O, inverted, then mounted on a glass microscope slide containing one drop of Vectashield anti-fade mounting media (Vector Laboratories). Coverslips were then covered with SuperMount (BioGenex, Fremont, CA) and allowed to dry overnight in the dark at 22°C. Cells were visualized on a Nikon TE2000 microscope (600X) equipped with a C1-plus confocal system with lasers at 402nm, 488nm and 561nm (Nikon, Melville, NY).

### Proximity ligation assay

Protein-protein interactions were verified using the proximity ligation assay (PLA, Duolink, orange for mouse/rabbit, Sigma/Aldrich). For these studies cells were fixed, permeabilized, blocked and exposed to primary antibodies as described above. All PLA reagents, dilution buffers, including wash buffers A and B, were provided by Sigma/Aldrich. After exposure to primary antibodies the coverslips were washed three times with TBST then PLA labeled secondary antibodies (anti-mouse, minus; anti-rabbit, plus) were added according to the manufacturer’s protocol. Briefly, to each coverslip 40μl of PLA antibody solution (8μl of each probe diluted in 24μl of blocking buffer) was added in a humidified incubator at 37°C for 1 h. After 1 h the coverslips were washed in wash buffer A twice for 5 min then 40μl of ligase solution (ligase diluted 1:40 in ligase dilution buffer), was added for 30 min at 37°C. After ligation the coverslips were washed twice (2 min) with wash buffer A then 40μl of polymerase solution (5μl polymerase diluted 1:10 in polymerase buffer) was added for 100 min at 37°C. After amplification the coverslips were washed twice with wash buffer B (10 min) then once with 0.01x wash buffer B (1 min). Coverslips were mounted as described in immunocytochemistry.

## Results

**Fig 1:**
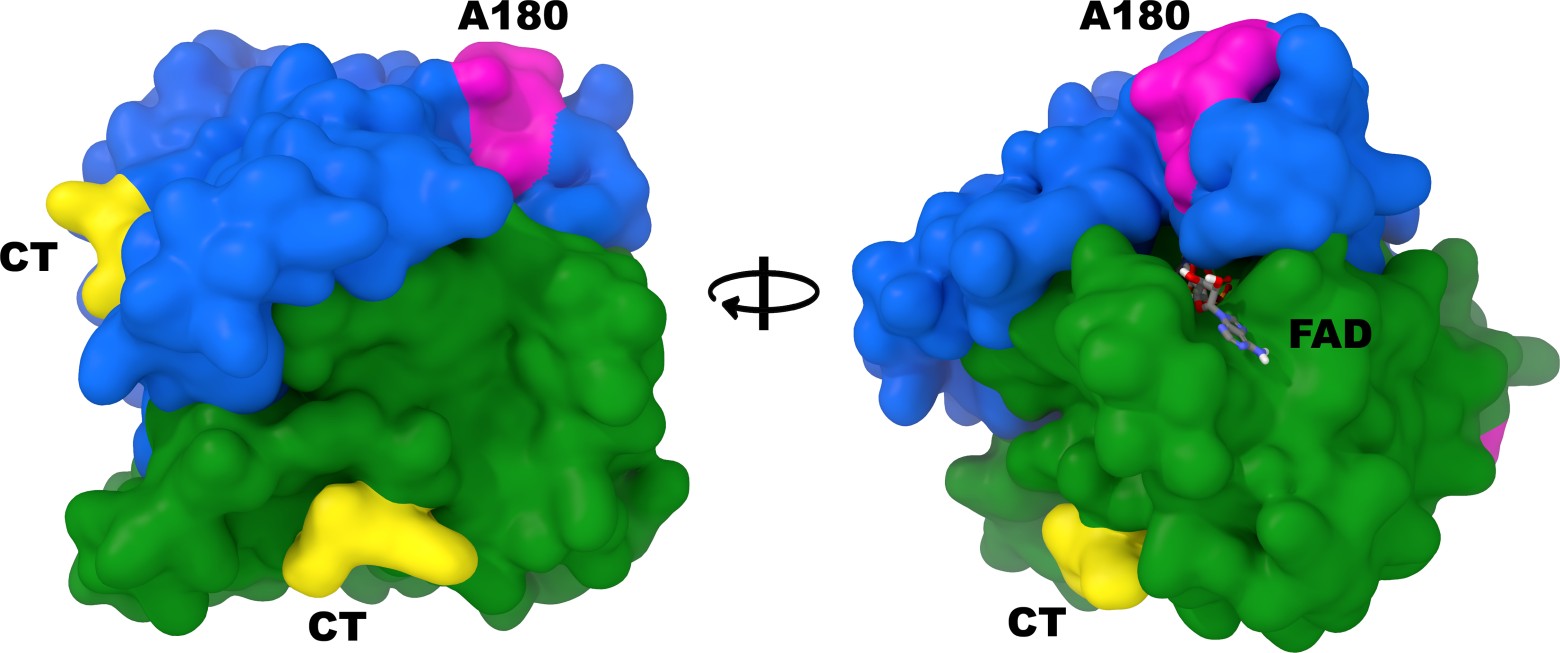
Relative positioning of the C-term and A180 antibody epitopes on human NQO1. Alternating viewpoints of the human NQO1 homodimer (PDB ID: 1D4A) with each monomer colored separately (blue and green) and the locations of the C-terminal epitopes (CT, yellow) and A180 epitopes (magenta) highlighted.

**Fig 2:**
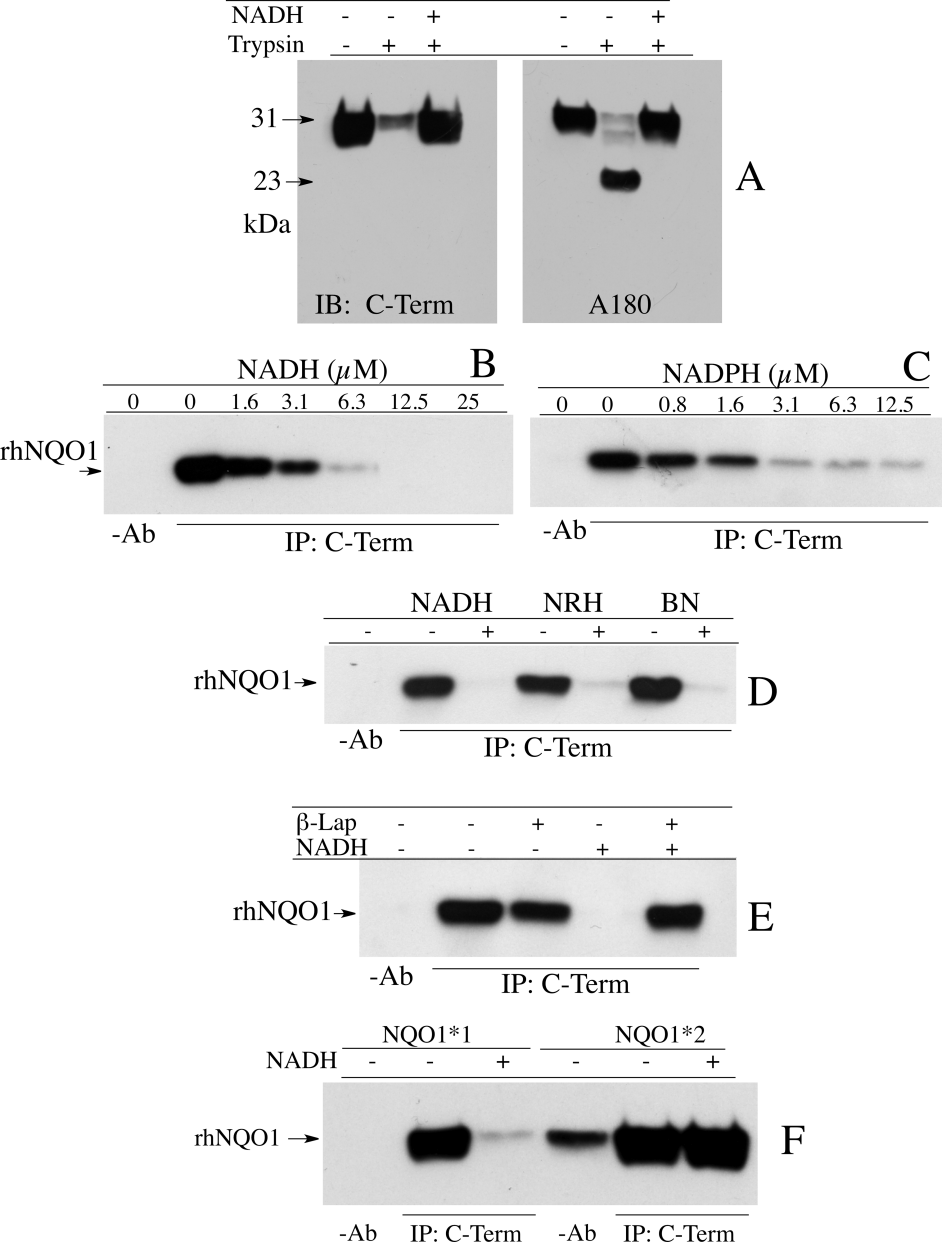
Reduced pyridine nucleotides induce a conformational change in NQO1 preventing antibodies from binding to the C-terminus. (A) Immunoblot analysis of rhNQO1 following incubation with trypsin in the absence and presence of NADH. (B, C) Immunoprecipitation of rhNQO1 by antibodies that target the C-terminal domain in the absence and presence of NADH or NADPH. (D) Immunoprecipitation of rhNQO1 by antibodies that target the C-terminal domain in the absence and presence of reduced nicotinamide analogs nicotinamide riboside (NRH) and benzyl dihydronicotinamide (BN). (E) Immunoprecipitation of rhNQO1 by antibodies that target the C-terminal domain following the oxidation of NADH by β-lapachone. (F) Immunoprecipitation of NQO1*1 and NQO1*2 proteins by antibodies that target the C-terminal domain in the absence and presence of NADH. Reaction conditions for immunoprecipitation studies are described in *Materials and methods*.

The work of Chen *et*. *al*. demonstrated that the C-terminus of the recombinant rat NQO1 homodimer was protected against proteolytic cleavage by reduced pyridine nucleotides (NADH or NADPH) [17]. We have extended these studies to show that the C-terminus of recombinant human NQO1 was also protected against tryptic cleavage by NADH. Immunoblot analysis of the tryptic digestion of rhNQO1 was carried out using two different anti-NQO1 antibodies. The C-terminal domain reactive antibody (C-term, rabbit polyclonal) was generated using a synthetic peptide corresponding to the last 13 amino acids (261 to 274) of human NQO1 while the A180 mouse monoclonal antibody was produced using full-length rhNQO1 and epitope-mapped to amino acids 67 to 79 on helix 7. The relative position of the C-term and A180 epitopes on the NQO1 homodimer is shown in Fig 1. Immunoblot analysis of the tryptic digestion of rhNQO1 using the C-term antibody showed that in the absence of NADH there was significant loss of full-length NQO1 (31kDa) but no detection of the major lower molecular weight cleavage product (23kDa, catalytic core domain), confirming that the C-terminus was lost during tryptic cleavage of oxidized NQO1 (Fig 2A, left panel). The addition of NADH protected human NQO1 against tryptic digestion and loss of the C-terminal domain (Fig 2A). Immunoblot analysis of the above reactions using the A180 antibody that targets helix 7 in the catalytic core domain confirmed that in the absence of NADH there was significant loss of full-length NQO1 (31kDa) with corresponding detection of the catalytic core domain at 23kDa (Fig 2A, right panel). These experiments demonstrate that upon binding reduced pyridine nucleotides human NQO1 may undergo a structural change that results in protection of the C-terminal domain against tryptic digestion.

If trypsin cannot access the C-terminal domain of reduced NQO1, then similarly, antibodies that target the C-terminus may also be excluded from binding to reduced NQO1. To examine whether the oxidized or reduced conformations of NQO1 could be distinguished by antibodies that bind to the C-terminus we performed immunoprecipitation studies using rhNQO1 under non-denaturing conditions in the absence and presence of increasing concentrations of NADH or NADPH (Fig 2B and 2C). Results from these studies clearly demonstrated that NQO1 could readily be immunoprecipitated by antibodies that target the C-terminal domain in the absence of NAD(P)H, but in the presence of NAD(P)H the immunoprecipitation of NQO1 was inhibited. These results extended to reducing co-factors which lack the dinucleotide structure of NAD(P)H including reduced nicotinamide riboside (NRH) and benzyl dihydronicotinamide (BN). Incubation of rhNQO1 with these reduced cofactors also prevented immunoprecipitation of NQO1 by antibodies that target the C-terminal domain (Fig 2D). To confirm the change in NQO1 structure was responsive to levels of reduced pyridine nucleotides and was reversible we incubated rhNQO1 with NADH and the quinone β-lapachone, which undergoes NQO1-dependent redox cycling resulting in the oxidation of NADH to NAD^+^. In these experiments the immunoprecipitation of NQO1 was inhibited in the presence of NADH but the addition of β-lapachone reestablished NQO1 immunoreactivity and the protein was efficiently pulled-down confirming that NQO1 alters its structure in response to the concentrations of reduced pyridine nucleotides (Fig 2E). To test the hypothesis that a structurally competent and catalytically active form of NQO1 is required to generate the change in protein conformation in response to NAD(P)H levels we performed similar immunoprecipitation studies using purified rhNQO1*2 which has a proline to serine amino acid substitution at position 187 and has poor catalytic activity due to its inability to bind the FAD cofactor efficiently [18]. The addition of NADH did not prevent the immunoprecipitation of the rhNQO1*2 by antibodies that target the C-terminal domain confirming the role of FAD and a reduced enzyme conformation in the loss of NQO1 immunoreactivity induced by reduced pyridine nucleotides (Fig 2F).

**Fig 3:**
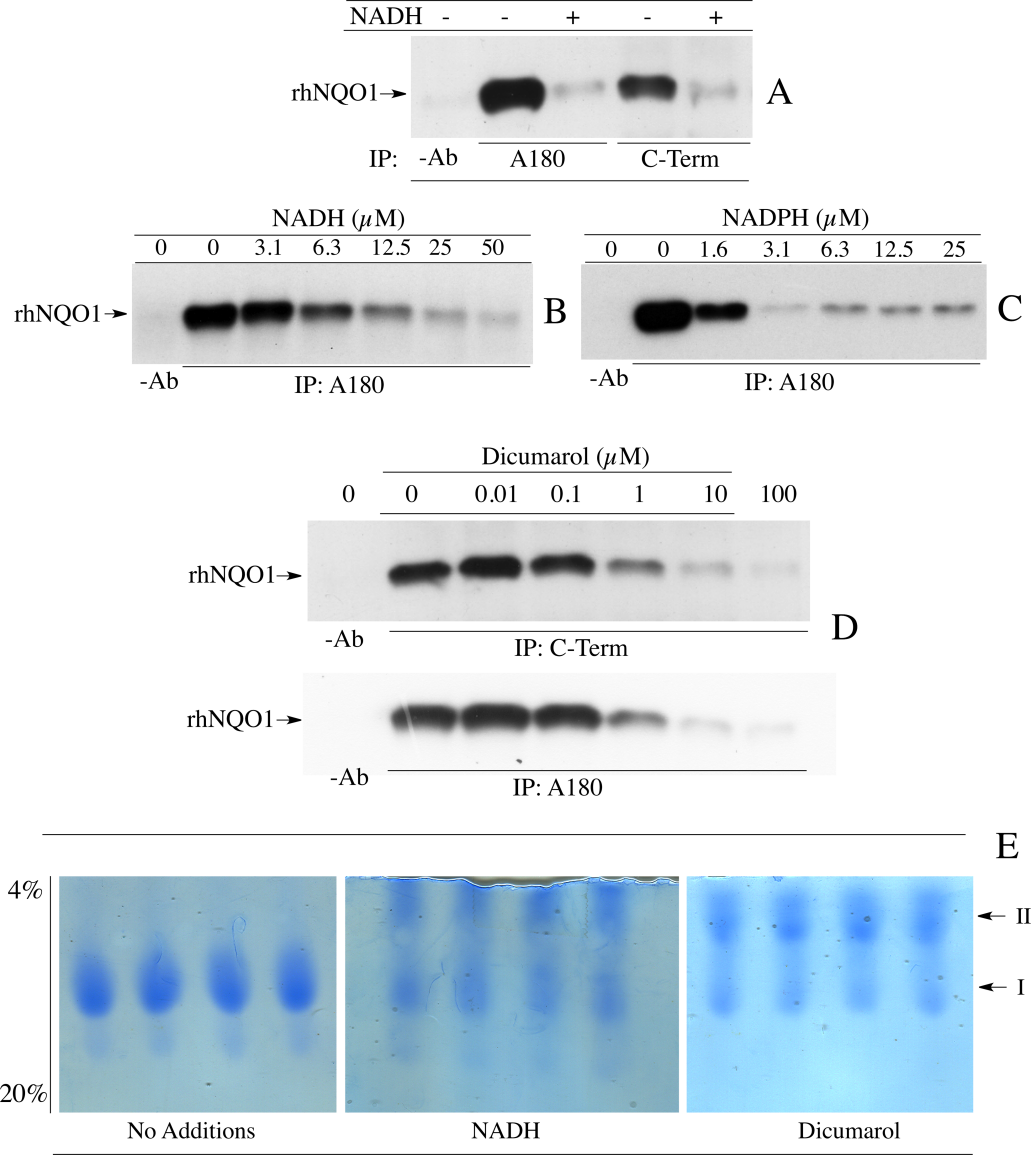
Reduced pyridine nucleotides and dicumarol induce a conformational change in NQO1. (A) Comparison of the ability of antibodies which bind to helix 7 (A180) and antibodies which bind to the C-terminal domain (C-Term) to immunoprecipitate rhNQO1 in the absence and presence of NADH. (B, C) Immunoprecipitation of rhNQO1 with the A180 antibody in the absence and presence of NADH or NADPH. (D) Immunoprecipitation of rhNQO1 by C-Term and A180 antibodies in the absence and presence of dicumarol. (E) The effect of NADH and dicumarol on the migration of rhNQO1 in non-denaturing PAGE. Reaction conditions for immunoprecipitation studies and non-denaturing PAGE are described in *Materials and methods*.

To determine if the structural change in NQO1 in response to NAD(P)H levels could be observed in other regions of NQO1 we performed similar experiments as those described in Fig 2, however, instead of using an antibody that targets the C-terminal domain of NQO1, we used a monoclonal antibody (A180) that binds to helix 7 in the catalytic core domain. Results from these studies showed that the A180 antibody was also prevented from immunoprecipitating rhNQO1 in the presence of reduced pyridine nucleotides (Fig 3A–3C). We extended these studies to the N-terminal domain of NQO1 using antibodies specific for this region. rhNQO1 was not immunoprecipitated by antibodies that bind to the N-terminal domain either in the absence or presence of NADH (data not shown). The inhibitor dicumarol, which also induces a conformational change in the active site of NQO1, has been shown to protect the C-terminal domain of rat NQO1 from proteolytic cleavage [17]. In our studies, the addition of dicumarol also prevented immunoprecipitation of rhNQO1 by C-term and A180 antibodies (Fig 3D). These data show that the loss of immunoreactivity in the presence of NAD(P)H or dicumarol extends beyond the C-terminal domain to also include helix 7 of the catalytic core domain. Interestingly, when rhNQO1 was analyzed by non-denaturing PAGE a slower migrating species (II) was observed when NADH or dicumarol was included in the loading and running buffers. This suggests that the formation of the slower migrating species may be related to loss of immunoreactivity (Fig 3E).

**Fig 4:**
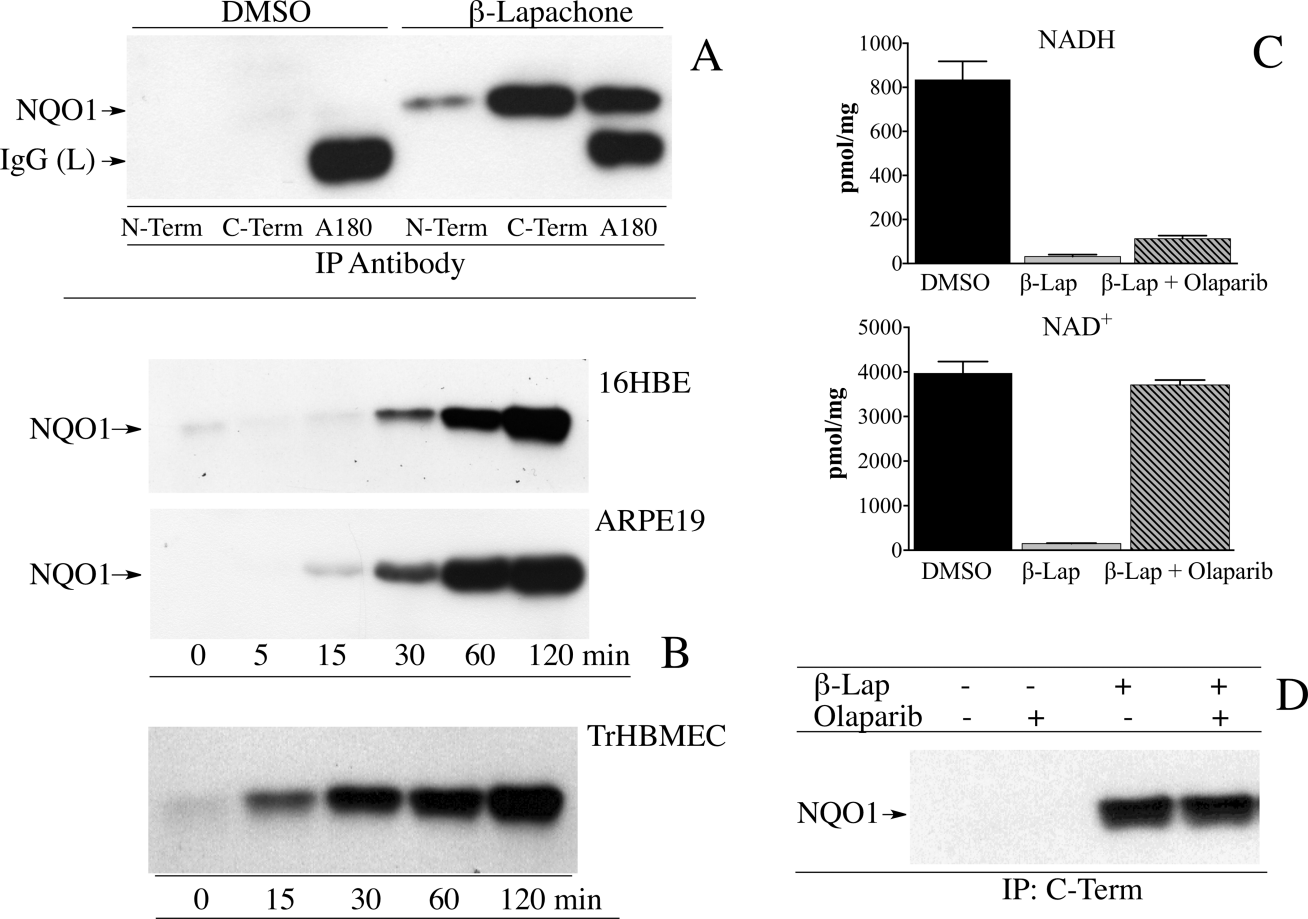
Intracellular oxidation of pyridine nucleotides induces a conformational change in NQO1. (A) 16HBE cells were treated with DMSO or β-lapachone (10μM) for 2 h after which NQO1 was immunoprecipitated using anti-NQO1 antibodies. (B) Human cell lines (16HBE, ARPE19, TrHBMEC) were treated with β-lapachone (10μM) for the indicated times after which NQO1 was immunoprecipitated using antibodies that recognize the C-terminal domain of NQO1. (C) Intracellular levels of NADH and NAD^+^ were measured by mass spectrometry in 16HBE cells treated with β-lapachone (10μM) for 2 h in the absence and presence of the PARP inhibitor olaparib (1μM). Results are the mean ± standard deviation, n = 3. (D) Immunoprecipitation of NQO1 from 16HBE cells treated with β-lapachone (10μM) in the absence and presence of olaparib (1μM) for 2 h. Reaction conditions are described in *Materials and methods*.

To validate the hypothesis that the structure of NQO1 changes in response to the intracellular NAD(P)^+^/NAD(P)H ratio we utilized human cell lines that express NQO1 and treated these cell lines with β-lapachone. In cell lines β-lapachone undergoes efficient redox cycling resulting in depletion of intracellular pools of reduced pyridine nucleotides [19]. Immunoprecipitation of NQO1 from human bronchial epithelial cells (16HBE) using antibodies directed against the N-terminus, C-terminal domain or helix 7 (A180) showed that in the absence of β-lapachone NQO1 could not be immunoprecipitated from whole cell lysates reflecting an abundant intracellular pool of reduced pyridine nucleotides, however, in cells treated with β-lapachone for 2 h NQO1 could be immunoprecipitated efficiently by either C-term or A180 antibodies. In agreement with studies using purified protein (above) NQO1 was not pulled-down from cell lysates by an antibody that target the N-terminus of NQO1 (Fig 4A). Time course studies in human cell lines expressing NQO1 (16HBE, ARPE-19 and TrHBMEC) showed that as the time of exposure to β-lapachone increased so did the amount of NQO1 that could be immunoprecipitated (Fig 4B). Treatment with β-lapachone did not increase the levels of NQO1 protein expression during the 2 h of exposure (S2 Fig). To confirm that treatment with β-lapachone results in oxidation of intracellular pools of NADH we measured NAD^+^ and NADH concentrations by mass spectrometry in 16HBE cells treated with either DMSO or β-lapachone for 2 h (Fig 4C). In these studies cells were also treated with β-lapachone and the PARP inhibitor olaparib to prevent PARP from consuming NAD^+^ generated from β-lapachone-induced oxidation of NADH. Treatment with β-lapachone resulted in near complete oxidation of intracellular pools of NADH, and as expected, PARP was responsible for consuming significant amounts of NAD^+^ since the addition of olaparib prevented NAD^+^ depletion (Fig 4C). Similar amounts of NQO1 were immunoprecipitated from 16HBE cells following β-lapachone treatment in the presence and absence of the PARP inhibitor olaparib confirming studies with rhNQO1 (above) that showed that reduced pyridine nucleotides are responsible for the loss of immunoreactivity of NQO1 (Fig 4D). These studies in human cells confirm studies using purified rhNQO1 and demonstrate that the immunoreactivity of NQO1 is dependent upon the NAD(P)^+^/NAD(P)H redox balance.

**Fig 5:**
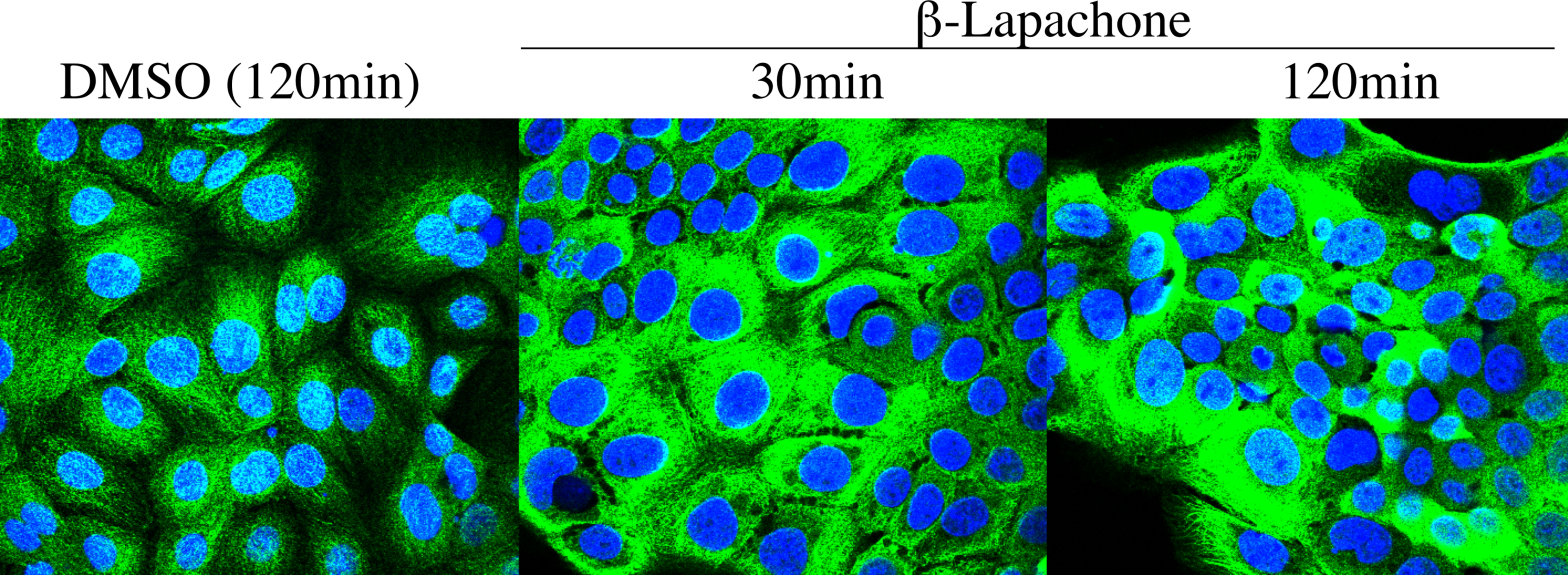
Increased immunostaining for NQO1 in 16HBE cells following treatment with β-lapachone. 16HBE cells were treated with DMSO or β-lapachone (10μM) for the indicated times then processed for immunocytochemistry and confocal analysis as described in *Materials and Methods*. Immunostaining for NQO1 was performed under non-denaturing conditions using the A180 antibody combined with DAPI nuclear staining.

Immunostaining for NQO1 under non-denaturing conditions offers another system in which to test the hypothesis that the immunoreactivity of NQO1 may be reflective of the pyridine nucleotide redox balance. Cellular regions with low levels of reduced pyridine nucleotides may demonstrate higher levels of NQO1 immunostaining reflecting the greater immunoreactivity of oxidized NQO1. To examine whether the immunoreactivity of NQO1 could be enhanced by decreasing the intracellular levels of reduced pyridine nucleotides 16HBE cells were treated with β-lapachone for the indicated times then processed for immunocytochemistry using very mild fixation (4% methanol-free formaldehyde, 0.1% Triton X-100), immunostained with the A180 antibody and then analyzed using confocal microscopy. Results from these experiments are shown in Fig 5 and clearly show a significant increase in the intensity of immunostaining for NQO1 with the A180 antibody in 16HBE cells following treatment with β-lapachone. These data show that the intensity of immunostaining for NQO1 increased as the intracellular levels of reduced pyridine nucleotides decreased confirming that the intensity of immunostaining for NQO1 is reflective of a more oxidized NAD(P)^+^/ NAD(P)H environment.

To confirm that in our immunocytochemical studies the A180 antibody targets only NQO1 and does not cross-react with other proteins we utilized siRNA to knockdown the levels of NQO1 in transformed human bone marrow endothelial cells (TrHBMEC) then carried out immunostaining using the A180 antibody. Results for these experiments are shown in S3 Fig and demonstrate that siRNA-mediated knockdown of NQO1 for 72 h eliminated immunostaining by the A180 antibody confirming that the immunostaining we observed was NQO1-specific.

**Fig 6:**
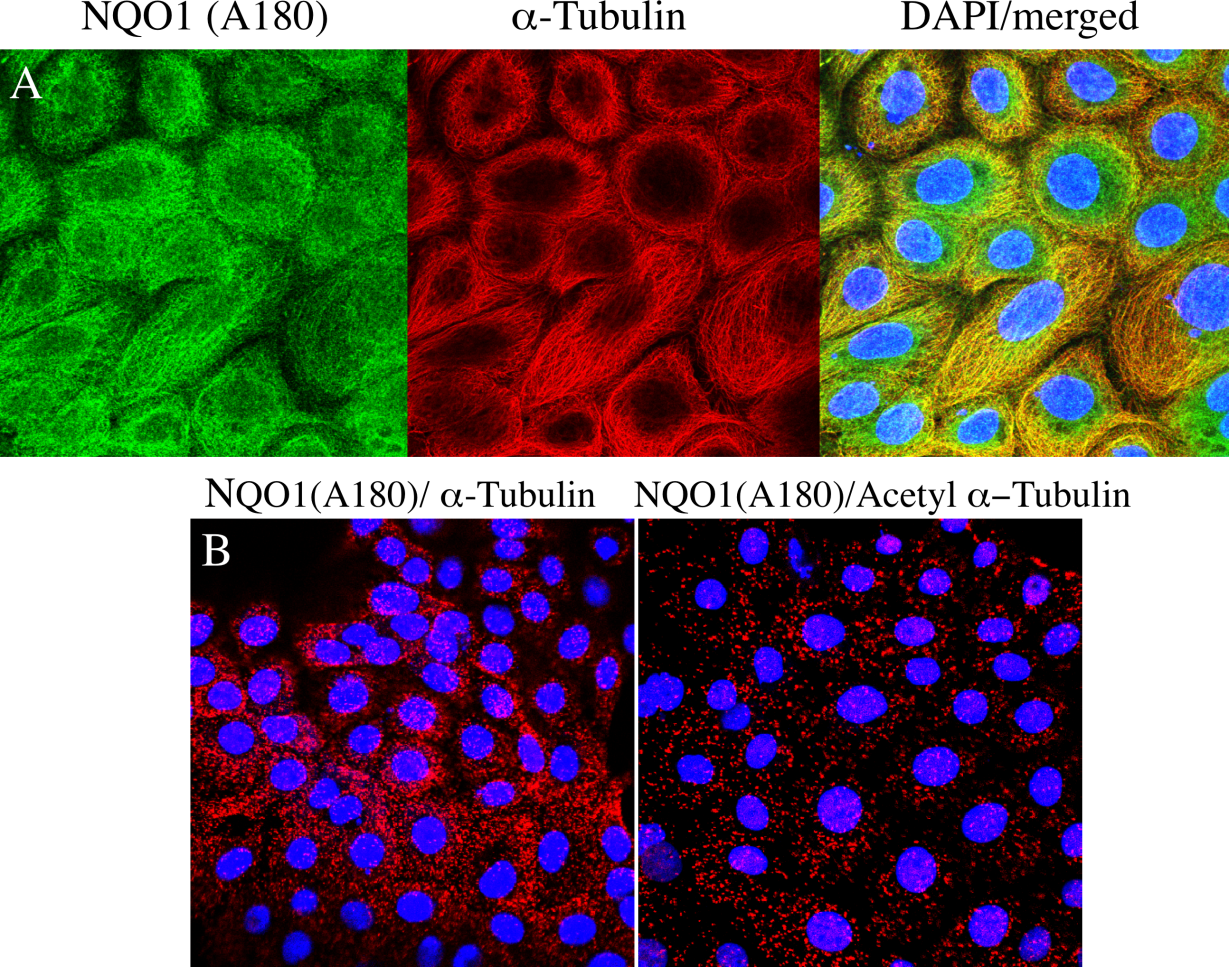
Co-localization of NQO1 with microtubules in 16HBE cells. Co-immunostaining for NQO1 (A180) and α-tubulin showing co-localization on microtubules. (A) Co-localization of NQO1 with α-tubulin using fluorescently-labeled secondary antibodies. (B) Co-localization of NQO1 with α-tubulin/acetyl α-tubulin using PLA-based detection. Immunostaining was performed as described in *Materials and methods*.

**Fig 7:**
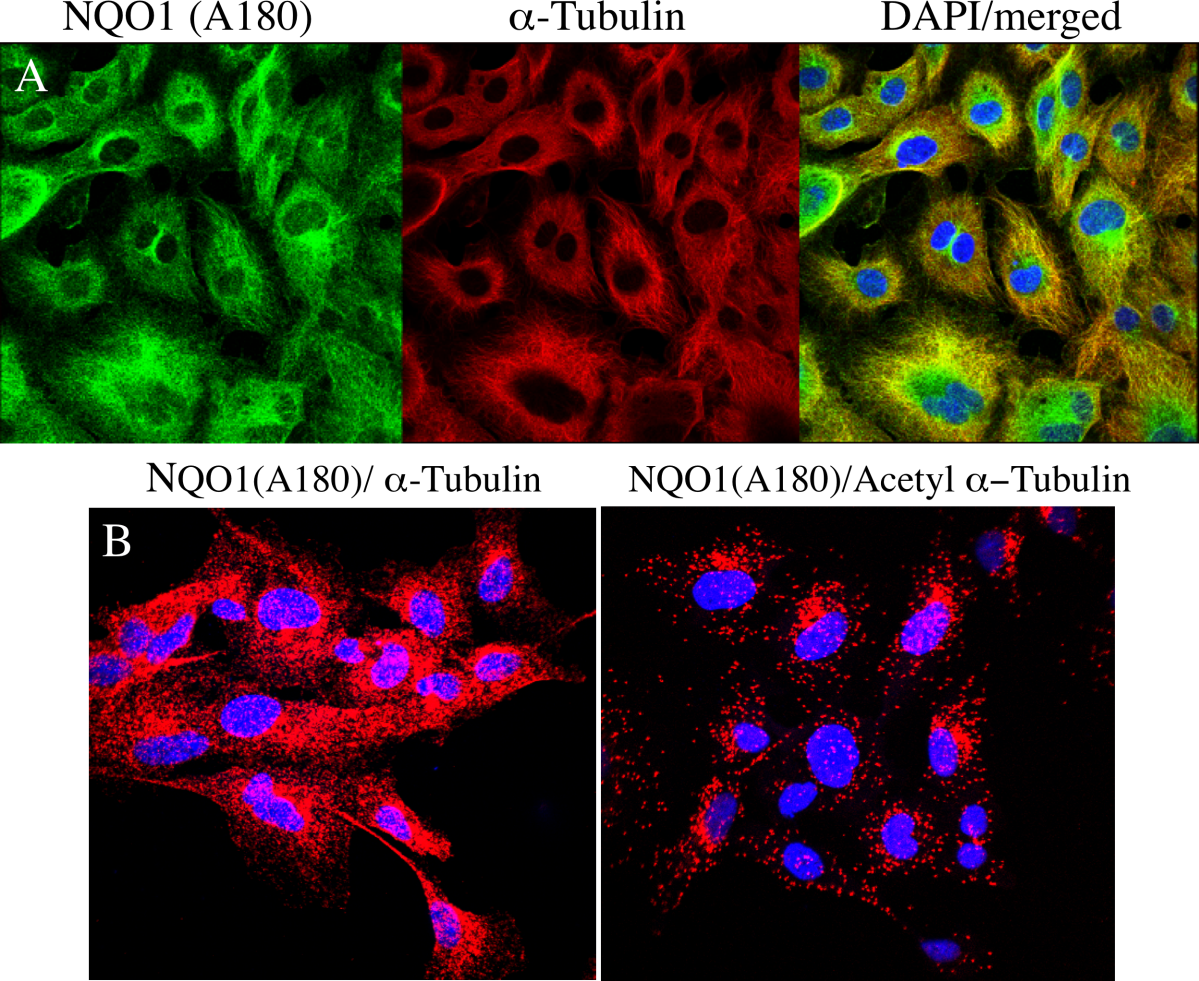
Co-localization of NQO1 with microtubules/acetylated microtubules in TrHBMEC. Co-immunostaining for NQO1 (A180) and α-tubulin showing co-localization on microtubules.(A) Co-localization of NQO1 with α-tubulin using fluorescently-labeled secondary antibodies. (B) Co-localization of NQO1 with α-tubulin/acetyl α-tubulin using PLA-based detection. Immunostaining was performed as described in *Materials and methods*.

**Fig 8:**
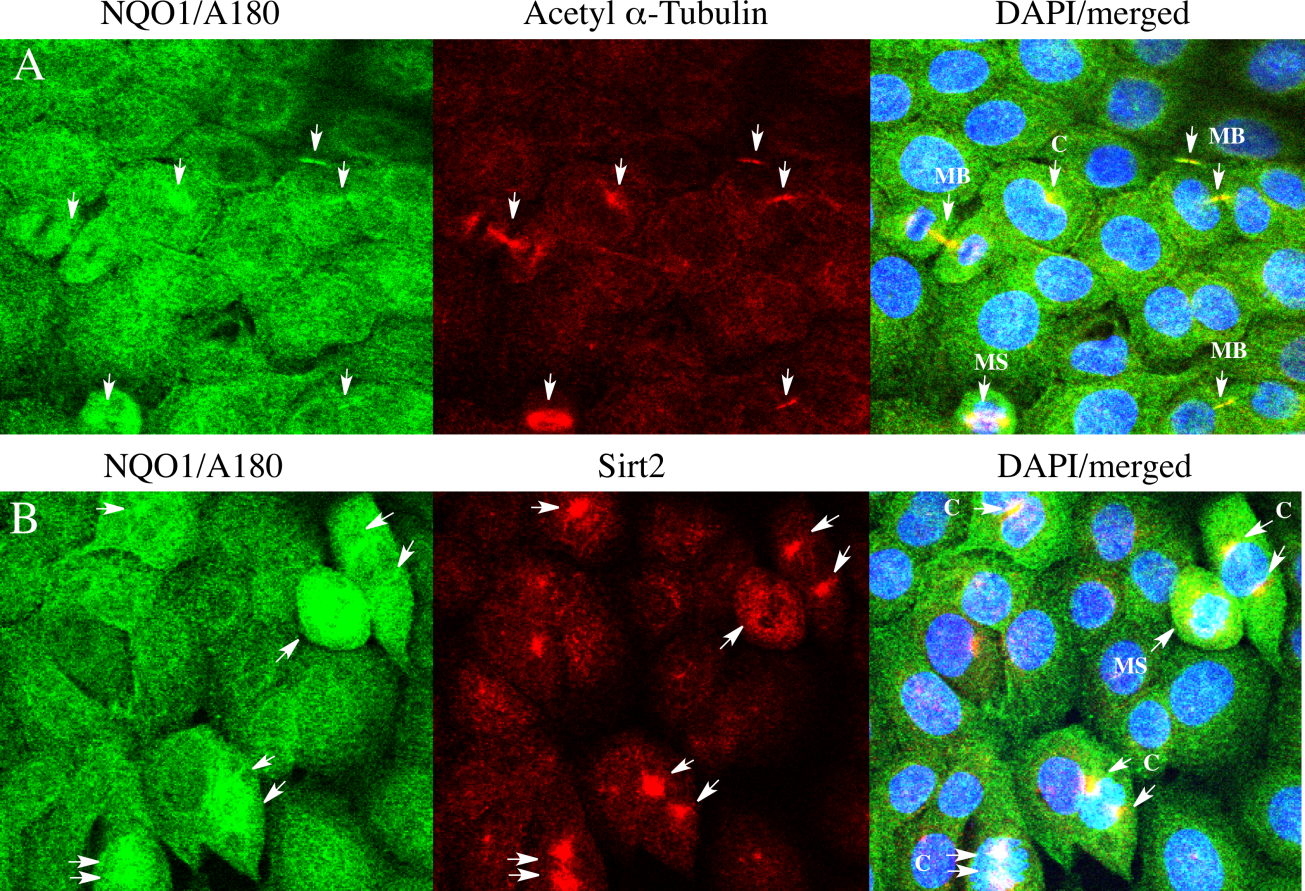
Co-localization of NQO1, Sirt2 and acetyl tubulin in 16HBE cells. (A) Co-immunostaining for NQO1 (green) and acetyl α-tubulin (red) showing co-localization on mitotic structures. (B) Co-immunostaining for NQO1 (green) and Sirt2 (red) showing co-localization on centrosome(s). Arrows indicate co-localization between high intensity immunostaining for NQO1, acetyl α-tubulin and Sirt2 in different stages of the centriole cycle. (C, centrosome(s); MS, mitotic spindles; MB, midbody).

**Fig 9:**
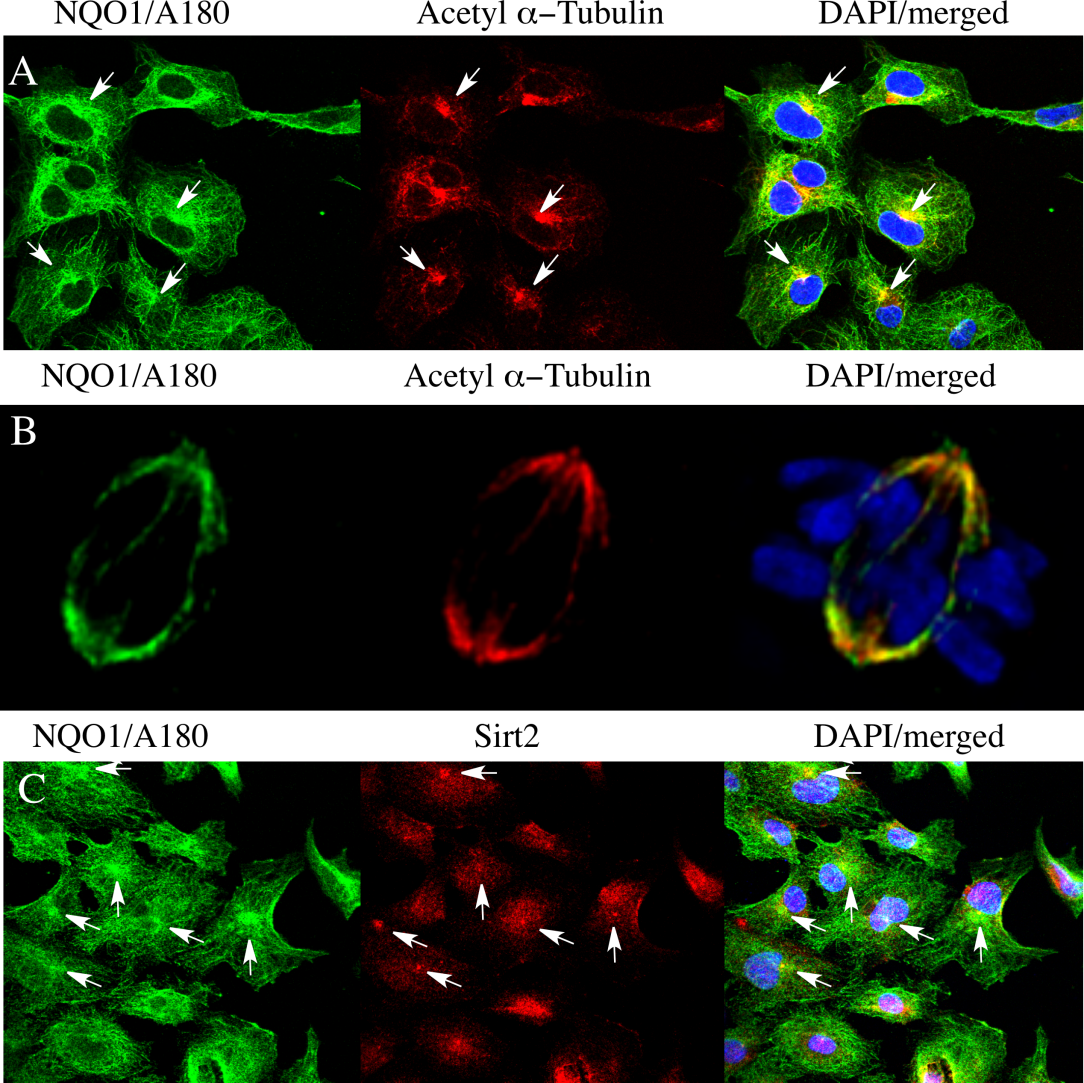
Co-localization of NQO1, acetyl α-tubulin and Sirt2 in TrHBMEC. (A) Co-immunostaining for NQO1 (A180, green) and acetyl α-tubulin (red) in TrHBMEC cells showing co-localization on centrosomes (arrows). (B) Co-immunostaining for NQO1 (A180, green) and acetyl α-tubulin (red) in TrHBMEC cells showing co-localization on mitotic spindles. Co-immunostaining for NQO1 (A180, green) and Sirt2 (red) in 16HBE cells. Arrows indicate co-localization between high intensity immunostaining for NQO1, acetyl α-tubulin and Sirt2 on the centrosomes.

Immunostaining for NQO1 using the A180 antibody was cytoskeleton-like in untreated 16HBE and TrHBMEC cells (Figs 6 and 7). Immunocytochemical studies using the A180 antibody and an antibody to α-tubulin demonstrated that NQO1 co-localized with microtubules in both cell types (Figs 6A and 7A). We also examined whether NQO1 was in close association with microtubules using the proximity ligation assay (PLA) with the anti-NQO1 (A180) antibody combined with either anti α-tubulin or anti acetyl α-tubulin (K40) antibodies. Under these conditions strong PLA signals were observed in 16HBE cells and TrHBMEC suggesting a close physical association between NQO1 and microtubules/acetylated microtubules (Figs 6B and 7B). In control reactions where either the A180, α-tubulin or acetyl α-tubulin antibody was substituted with a species and isotype-matched control antibody only minimal PLA signals were detected (S4 Fig). Interestingly, high intensity immunostaining for NQO1 was observed to co-localize with acetylated α-tubulin in the perinuclear regions near centrosomes in both cell lines (Figs 8A and 9A). Another important finding was the co-localization of high intensity immunostaining for NQO1 and acetylated α-tubulin on the mitotic spindles and midbody region in 16HBE and TrHBMEC cells (Figs 8A and 9B). A possible link between NQO1 and acetylated microtubules is the NAD^+^-dependent deacetylase Sirtuin 2 (Sirt2). Co-immunostaining for NQO1 (A180) and Sirt2 also showed a high intensity signal for NQO1 co-localizing with Sirt2 on the centrosome(s) in16HBE and TrHBMEC cells (Figs 8B and 9C). Immunostaining for Sirt2 on the centrosome was confirmed by co-localization with γ-tubulin (S5 Fig). These data demonstrate that high intensity immunostaining for NQO1 co-localizes with (I) acetyl α-tubulin and Sirt2 on the centrosome(s) and (II) with acetyl α-tubulin on the mitotic spindles and midbody. These data show potential links between pyridine nucleotide redox balance, NQO1, acetylated microtubules and Sirt2 particularly during the centriole duplication cycle in mitosis.

## Discussion

These data demonstrate that upon binding reduced pyridine nucleotides, NQO1 undergoes a change in structure resulting in loss of immunoreactivity to epitopes located in both the catalytic core and C-terminal domains. Antibodies that target epitopes located in helix 7 in the catalytic core domain (A180) or epitopes in the C-terminal domain (C-term) do not bind NQO1 in the presence of reduced pyridine nucleotides or the inhibitor dicumarol. Since NQO1 has a single binding site for both reduced pyridine nucleotides and substrates it has been proposed that the active site changes conformation after hydride transfer to FAD to expel the oxidized pyridine nucleotide and promote substrate binding [13, 20]. X-ray crystal structures for NQO1 have been obtained with oxidized pyridine nucleotides bound into the active site [21]; however, no structures have been published with NQO1 in the reduced conformation hence preventing a direct comparison between the two structures. The ability of dicumarol to mimic reduced pyridine nucleotides and induce a change in the conformation of NQO1 is important since a x-ray crystal structure has been published with dicumarol bound into the active site of NQO1 [22]. From this study the authors concluded that the most prominent change in the conformation of NQO1 induced by dicumarol binding involved Tyr128 and Phe 232 located on the surface of the catalytic pocket [22]. The ability of dicumarol to alter the active site of NQO1 supported earlier studies using artificial flavins which concluded that dicumarol binding induced a conformational change in the FAD-binding pocket of NQO1 [23].

Interestingly, neither the C-terminal domain or helix 7 have been shown to directly participate in the binding of FAD, reduced pyridine nucleotides or substrates suggesting that the loss of these epitopes may reflect a much greater change in the conformation of NQO1 to include regions outside the active site. Alternatively, the change in the conformation of NQO1 following binding of reduced pyridine nucleotides or dicumarol could promote the association of two homodimers to form a tetramer. When rhNQO1 was analyzed under non-denaturing PAGE in the presence of NADH or dicumarol a much slower migrating species was generated suggesting the possibility of tetramer formation. The association of two homodimers of reduced NQO1 to form a tetramer could result in the simultaneous blockage of epitopes on the C-terminal and the catalytic core domains. The ability of NQO1 to form tetramers has been proposed previously to explain data obtained from analytical ultracentrifugation experiments using rat NQO1 [24]. In these experiments it was proposed that, like many other reductases [22], NQO1 may exist in equilibrium between a homodimer and tetramer. Whatever the mechanism the data presented in this manuscript using antibodies supports the hypothesis that the NAD(P)^+^/NAD(P)H redox balance alters the structure of NQO1 to promote the binding or disassociation of NQO1 with itself, other proteins or mRNA.

The ability of antibodies to distinguish between oxidized and reduced conformations of NQO1 is intriguing and raises the possibility that this difference in epitope recognition in NQO1 may be exploited under non-denaturing conditions including immunocytochemical studies to detect regions within the cell with a more oxidized pyridine nucleotide microenvironment. Immunostaining of human cells for NQO1 with the A180 antibody demonstrated regions with higher intensity staining and these areas co-localized with acetylated microtubules and the NAD^+^-dependent deacetylase Sirt2. The association between NQO1, acetylated microtubules and Sirt2 was observed most predominantly throughout the centriole duplication cycle in mitosis. Previous studies have shown NQO1 immunoreactivity on centrosomes, mitotic spindles and the midbody [25, 26] while acetylated α-tubulin and Sirt2 co-localization has been detected on the centrosome, mitotic spindles and the midbody [27–29], but to our knowledge, this is the first report of co-localization of NQO1, acetylated microtubules and Sirt2. The dynamic state of microtubule acetylation-deacetylation is controlled primarily by α-tubulin acetyltransferase 1 (acetylation) and HDAC6 and Sirt2 (deacetylation) [30–33]. These results raise the possibility that NQO1 may influence the dynamic balance of acetylation–deacetylation of microtubules especially during mitosis by providing NAD^+^ for Sirt2-mediated deacetylase activity in close proximity to microtubules. Through the consumption of NAD^+^, Sirt2 could generate a pyridine nucleotide-depleted microenvironment around acetylated microtubules to which NQO1 responds by adopting an oxidized conformation promoting NQO1 binding to microtubules/acetylated microtubules. The depletion of pyridine nucleotide pools near microtubules may be enhanced by PARP, which also consumes NAD^+^, and has been observed to localize to the centrosome(s) during mitosis [34, 35]. We speculate that binding of oxidized NQO1 to acetylated microtubules could act as a mechanism to retain the enzyme near microtubules where it would be beneficial since NQO1 could provide both antioxidant protection and a source of NAD^+^.

In summary, we have presented data demonstrating that upon binding reduced pyridine nucleotides or dicumarol NQO1 undergoes a change in structure which results in the loss of antibody binding to epitopes located in both the catalytic core and C-terminal domains. The ability of reduced pyridine nucleotides to suppress NQO1 immunoreactivity predicts that in immunocytochemical studies under non-denaturing conditions, high intensity staining for NQO1 is reflective of an oxidized pyridine nucleotide microenvironment. In cells, high intensity immunostaining for NQO1 co-localized with acetylated microtubules and Sirt2 suggesting that NQO1 may be involved in the regulation of microtubule acetylation. These data support a role for NQO1 as a redox-dependent switch where the protein responds to the NAD(P)^+^/NAD(P)H redox environment by altering its structure promoting the binding or dissociation of NQO1 with target macromolecules.

## Supporting information

S1 Fig Purity of purified rhNQO1 was assessed by 12% SDS-PAGE with Coomassie dye R-250 staining. (TIFF)

S2 Fig NQO1 protein expression following β-lapachone treatment. NQO1 protein expression was measured by immunoblot analysis in cell lysates (20μg) from cells treated with β-lapachone (10μM) for the indicated times. β-Actin was included as a loading control.
(TIF)

S3 Fig siRNA-mediated knockdown of NQO1 eliminates immunostaining of NQO1 by the A180 antibody. TrHBMEC were treated with either non-targeting siRNA (left panels) or siRNA targeting NQO1 (right panels) for 72 h after which the cells were immunostained for NQO1 using the A180 antibody. siRNA treatments and immunostaining were performed as described in *Materials and methods*.
(TIFF)

S4 Fig Control studies for PLA-based detection of NQO1 co-localization with α-tubulin and acetyl α-tubulin. Immunocytochemical staining using PLA detection in 16HBE cells and TrHBMEC where one of the primary antibody pairs were substituted with a species and isotype-matched control antibody.
(TIFF)

S5 Fig Co-localization of Sirt2 with the centrosome marker γ-tubulin. 16HBE cells were co-immunostained with Sirt2 and γ-tubulin antibodies. Arrows indicate co-localization of Sirt2 and γ-tubulin on the centrosomes of 16HBE cells.
(TIFF)

S1 Table Antibodies and dilutions used in this study. (DOCX)
