# Supplementary figures and images for: Redox modulation of NQO1
Source: PLoS One. 2018 Jan 3;13(1):e0190717. doi: 10.1371/journal.pone.0190717 (PMC5752044; doi:10.1371/journal.pone.0190717)

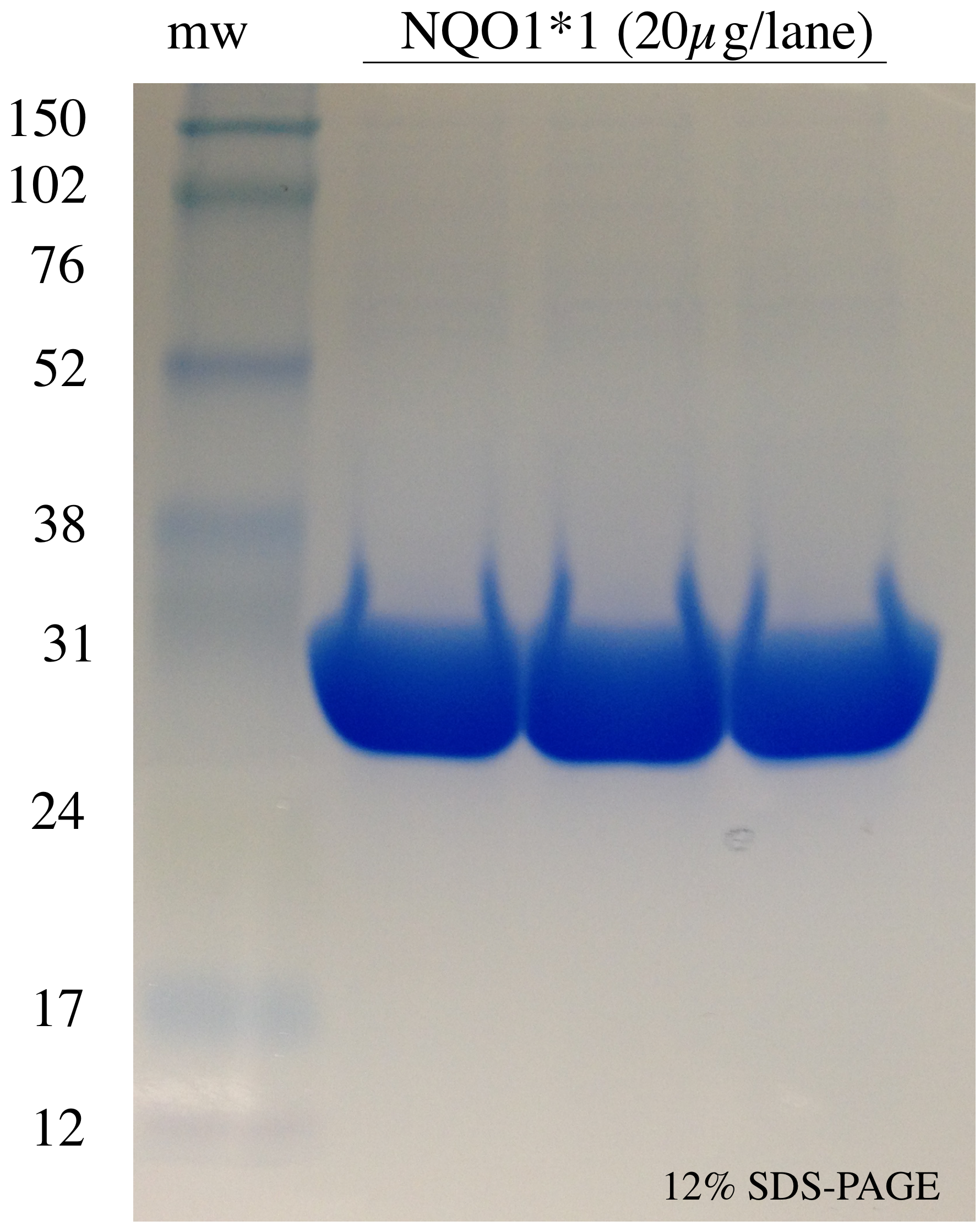

Supplement: S1 Fig — (TIFF) [file pone.0190717.s001.tiff]

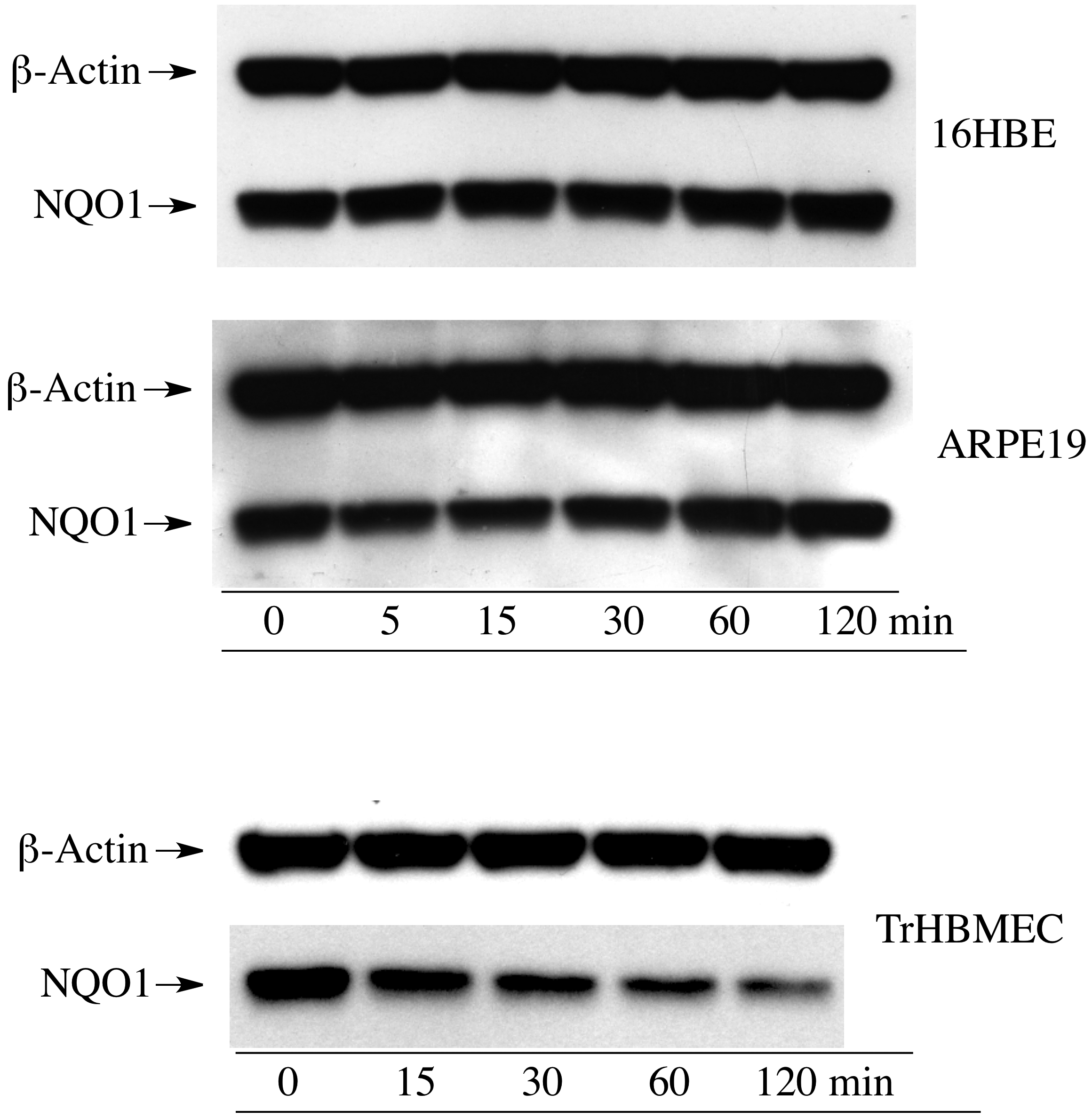

Supplement: S2 Fig — NQO1 protein expression was measured by immunoblot analysis in cell lysates (20μg) from cells treated with β-lapachone (10μM) for the indicated times. β-Actin was included as a loading control. (TIF) [file pone.0190717.s002.tif]

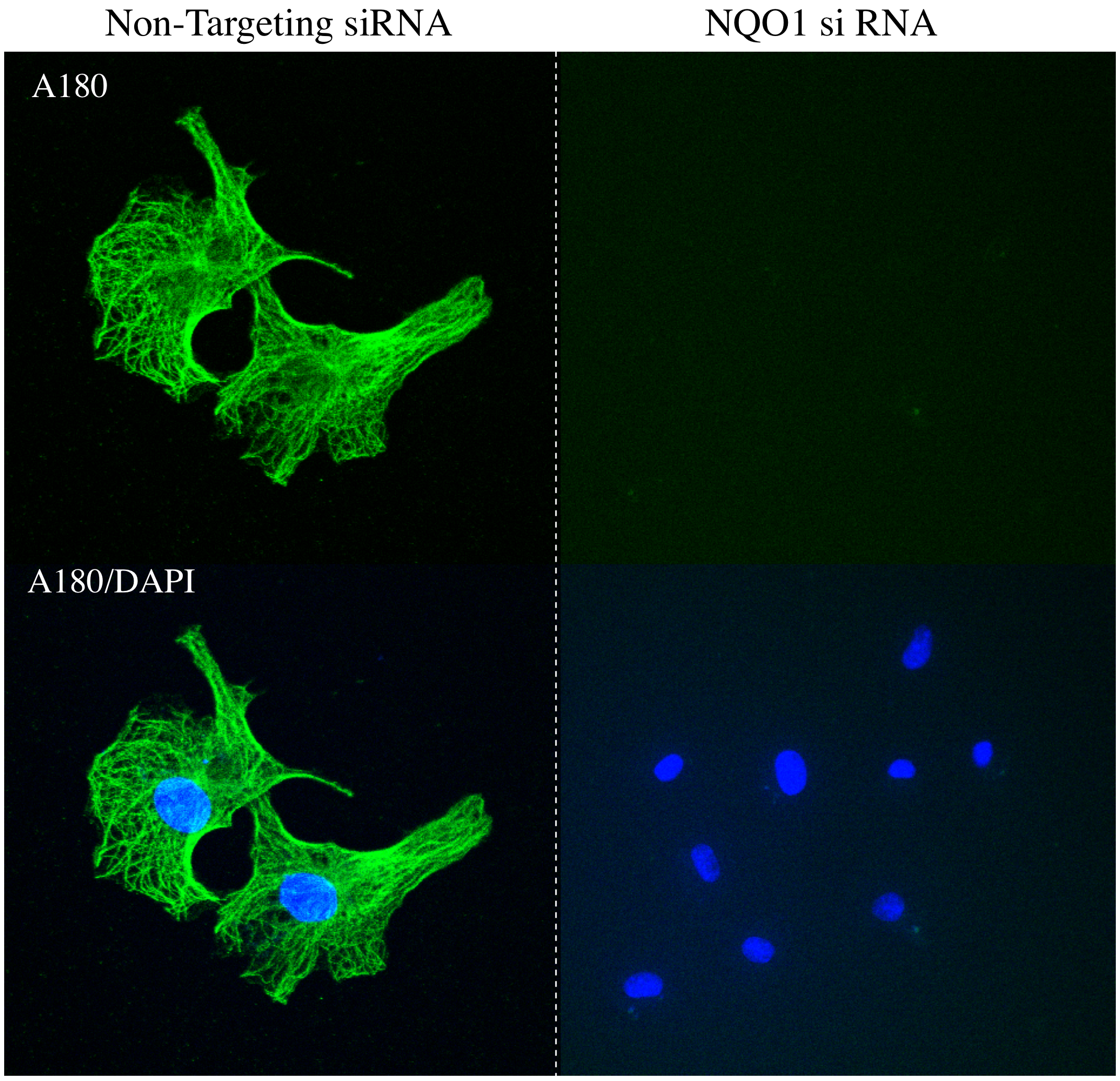

Supplement: S3 Fig — TrHBMEC were treated with either non-targeting siRNA (left panels) or siRNA targeting NQO1 (right panels) for 72 h after which the cells were immunostained for NQO1 using the A180 antibody. siRNA treatments and immunostaining were performed as described in Materials and methods. (TIFF) [file pone.0190717.s003.tiff]

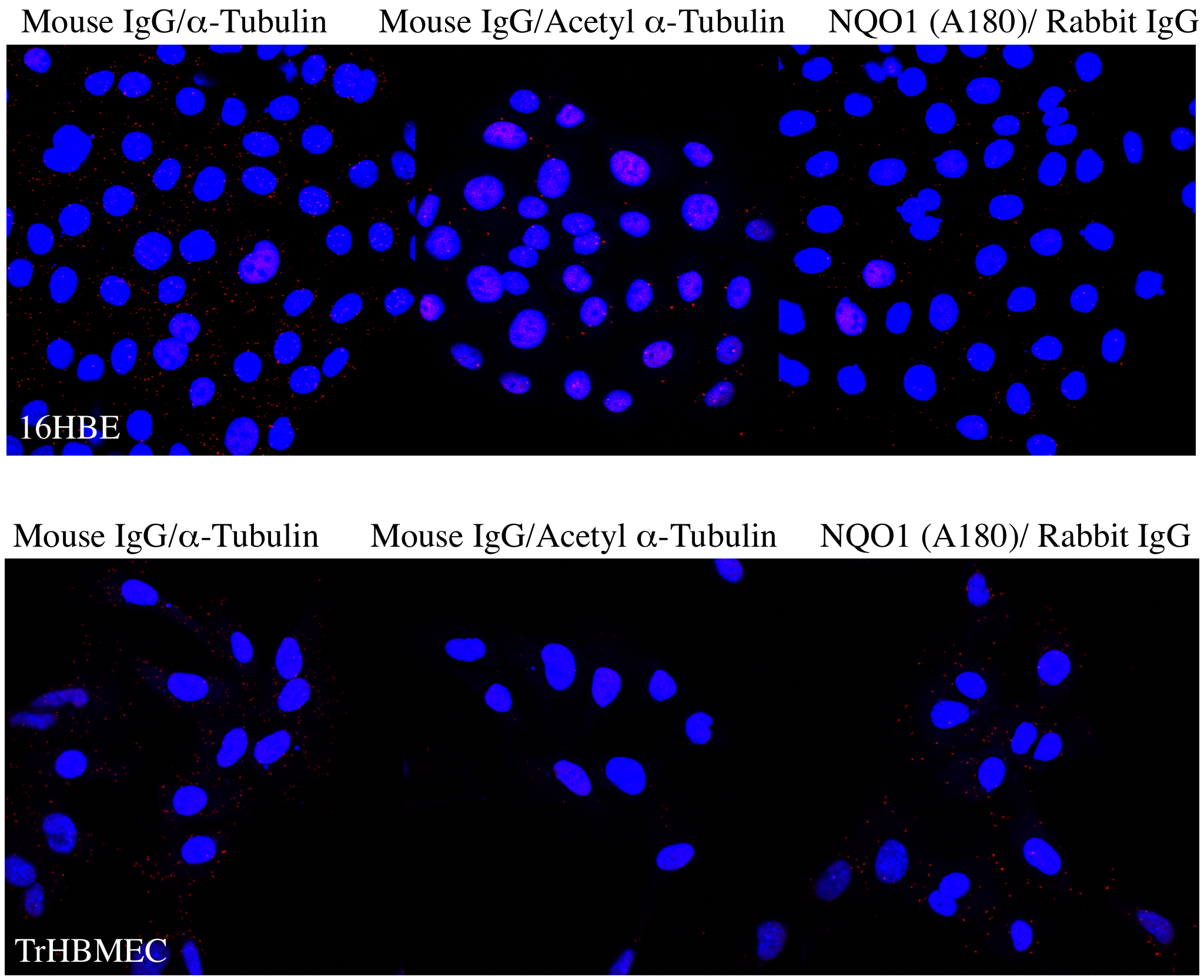

Supplement: S4 Fig — Immunocytochemical staining using PLA detection in 16HBE cells and TrHBMEC where one of the primary antibody pairs were substituted with a species and isotype-matched control antibody. (TIFF) [file pone.0190717.s004.tiff]

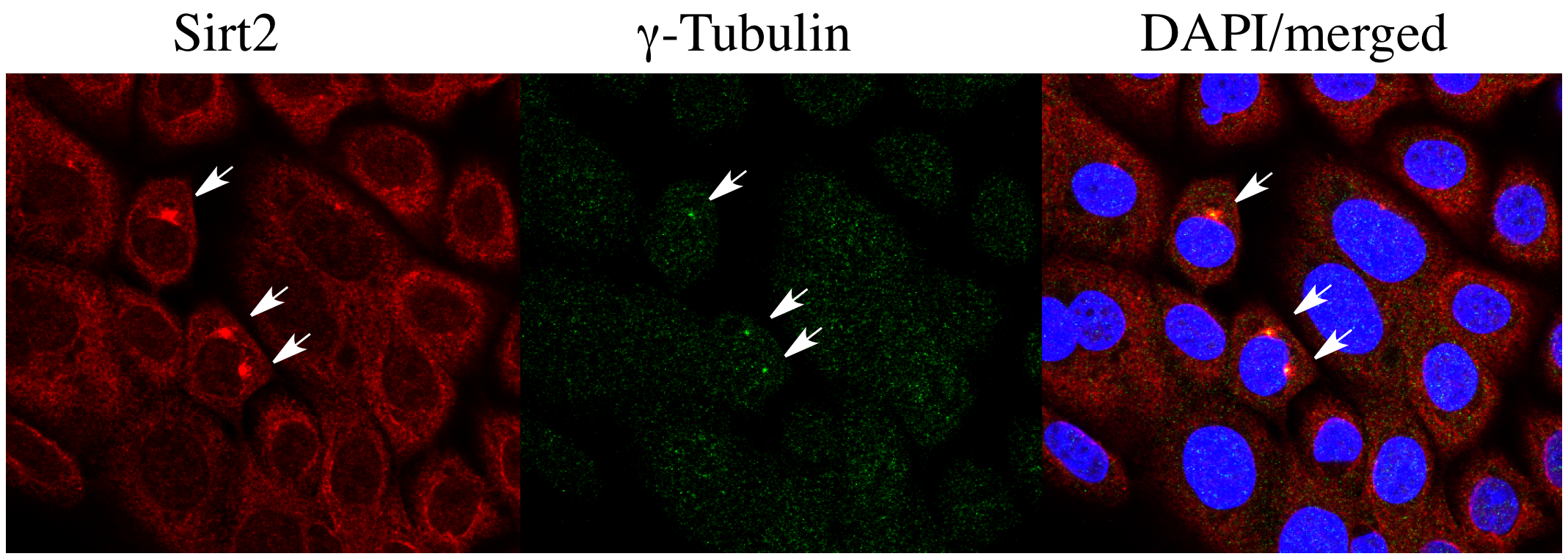

Supplement: S5 Fig — 16HBE cells were co-immunostained with Sirt2 and γ-tubulin antibodies. Arrows indicate co-localization of Sirt2 and γ-tubulin on the centrosomes of 16HBE cells. (TIFF) [file pone.0190717.s005.tiff]
