# Supplementary material for: Redox modulation of NQO1
Source: PLoS One. 2018 Jan 3;13(1):e0190717. doi: 10.1371/journal.pone.0190717 (PMC5752044; doi:10.1371/journal.pone.0190717)
Supplement: S1 Table — (DOCX) [file pone.0190717.s006.docx]

S1 Table. Antibodies and dilutions used in this study

| **Antibody** | **Catalog Number** | **Source** | **Type** | **Dilution** | **Time/ Application** |
| --- | --- | --- | --- | --- | --- |
| NQO1 (A180) | NB200-209 | Novus Biologicals | mouse mAb | 1:500-1:1000 | 1h ^1^(IP, IB, ICC) |
| NQO1 (C-Term) | N5288 | Sigma/Aldrich | rabbit polyclonal | 1:1000-1:2000 | 1h (IP, IB) |
| α-Tubulin | Ab18251 | Abcam | rabbit polyclonal | 1:1000 | 1h (ICC) |
| Acetyl α-Tubulin (K40) | 5335 | Cell Signaling | rabbit mAb | 1:500 | 1h (ICC) |
| Sirt2 | S8447 | Sigma/Aldrich | rabbit polyclonal | 1:1000 | 1h (ICC) |
| γ-Tubulin | NB600-412 | Novus  Biologicals | mouse mAb | 1:250 | 16h (ICC) |
| β-Actin | A2228 | Sigma/Aldrich | mouse mAb | 1:5000 | 0.5h (IB) |

^1^IP, immunoprecipitation; IB, immunoblot; ICC, immunocytochemistry.
